# Comparing Methods of Feature Extraction of Brain Activities for Octave Illusion Classification Using Machine Learning

**DOI:** 10.3390/s21196407

**Published:** 2021-09-25

**Authors:** Nina Pilyugina, Akihiko Tsukahara, Keita Tanaka

**Affiliations:** 1 Graduate School of Advanced Science and Technology, Tokyo Denki University, Hiki-gun, Saitama 350-0394, Japan; 2 Graduate School of Science and Engineering, Tokyo Denki University, Hiki-gun, Saitama 350-0394, Japan; tsukahara@mail.dendai.ac.jp (A.T.); ktanaka@mail.dendai.ac.jp (K.T.)

**Keywords:** feature selection, machine learning, octave illusion, auditory illusion, MEG

## Abstract

The aim of this study was to find an efficient method to determine features that characterize octave illusion data. Specifically, this study compared the efficiency of several automatic feature selection methods for automatic feature extraction of the auditory steady-state responses (ASSR) data in brain activities to distinguish auditory octave illusion and nonillusion groups by the difference in ASSR amplitudes using machine learning. We compared univariate selection, recursive feature elimination, principal component analysis, and feature importance by testifying the results of feature selection methods by using several machine learning algorithms: linear regression, random forest, and support vector machine. The univariate selection with the SVM as the classification method showed the highest accuracy result, 75%, compared to 66.6% without using feature selection. The received results will be used for future work on the explanation of the mechanism behind the octave illusion phenomenon and creating an algorithm for automatic octave illusion classification.

## 1. Introduction

The auditory illusion is a false perception of real auditory stimuli. Unlike hallucinations, which have no sensory base, auditory illusions are always caused by external stimuli. Compared with visual illusions, people who perceive auditory illusions are not always aware of them. It is difficult for the human brain to separate the real and perceived sounds. In addition to physical pathologies, the ability to classify them depends on the mental status of the subject. Not all auditory illusions are symptoms of psychological disorders; the main characteristics of pathological illusions are their connection with the subject’s painful experiences and worries and the absence of the context of the situation. Auditory illusions can accompany depression, panic disorders, delirium, and other mental problems [1]. Therefore, understanding the mechanisms underlying auditory illusions will contribute to our knowledge of pathological mental issues.

**Figure 1:**
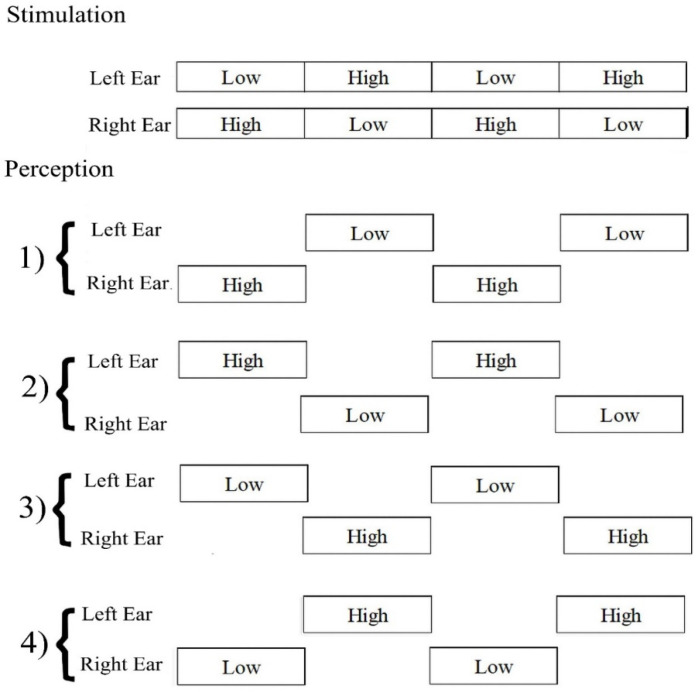
Octave illusion stimulation and four perception patterns.

The octave illusion is one of the less-studied types of auditory illusions. It is induced by two dichotic sounds (400 and 800 Hz) played simultaneously and constantly in both ears [2]. The main characteristic of this phenomenon is the perception that occurs only in one ear at a time. A low tone presented to the right ear and a high tone to the left ear can be perceived in four main patterns. Perception is described as a single high-pitch tone in one ear, alternating with a single low-pitch one in another ear (Figure 1).

The behavioral explanation for octave illusion is the “what” and “where” model, in which “what” is a perceived sound determined by the dominant ear and “where” is a sound location defined in the ear receiving a high tone [3].

However, the difference in brain responses between subjects who experience the illusion and those who do not is unclear. Although it has been proven that the pitch of the illusion perception has a main neural counterpart bilaterally in Heschl’s gyrus (primary auditory cortex), the processes underlying the octave illusion have not been clarified yet. We suggest that it is possible to separate illusion (ILL) and nonillusion (non-ILL) groups based on the difference in auditory steady-state responses (ASSRs) using machine learning methods.

Machine learning has a wide application in the biomedical field. It is being used for EEG-based BCI for classification person intentions [4], gait decoding [5], or short/zero-calibration calibration [6]. Along with EEG data, machine learning is being used for the analysis of fMRI data [7] and MEG data. There are studies dedicated to applying deep learning to MEG data source localizations [8] and decoding signals [9]. Machine learning has also proved itself as a powerful tool for recognizing subtle patterns in complex data, such as ASSR [10,11].

ASSRs are auditory evoked potentials that arise in response to rapid auditory stimulation. They can be used as a measurement to estimate the brain’s ability to generate responses, which can be used to differentiate subjects with normal and pathological hearing sensitivity [12]. Pathological hearing sensitivity often corresponds with mental diseases, such as schizophrenia, and researching ASSRs can help understand these problems as well.

**Figure 2:**
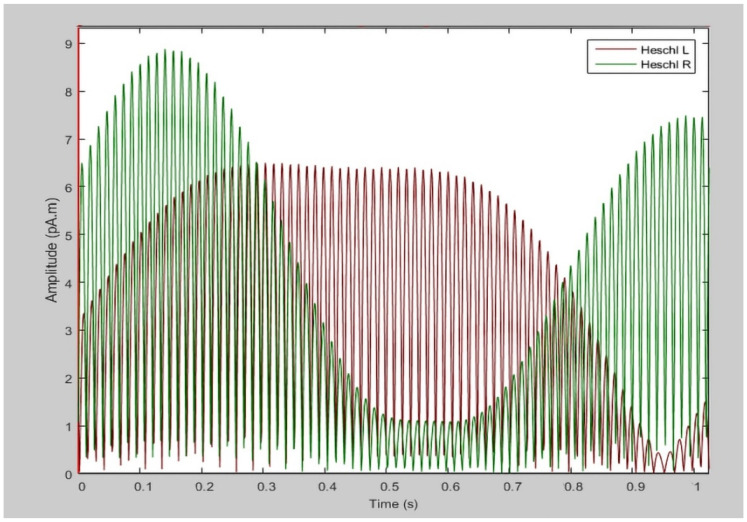
The random example of ASSR data used in this study.

**Figure 3:**
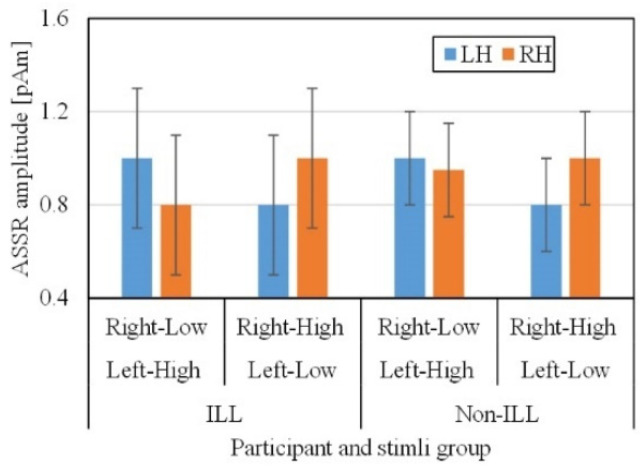
ASSR amplitude in nonillusion and illusion groups. RH and LH show the ASSR amplitude in the right and left hemispheres.

In this study, there was no evident gap between the average ASSR responses (Figure 2) of the ILL and non-ILL groups during the octave illusion stimulation (Figure 3). Therefore, it is impossible to distinguish them by simple comparison, and we hypothesized that the selected features of ASSR patterns of the left and right hemispheres would provide enough information for binary classification. However, the usual process of data selection, when certain features are added or removed individually depending on the results, is more difficult to implement for this task because of the lack of information about the octave illusion. Because we do not know what exactly defines the octave illusion, limiting the size of the dataset risks losing valuable features without resolving problems with overfitting or improving accuracy. Therefore, we investigated how the use of automatic feature selection accomplishes octave illusion classification using machine learning.

In machine learning, gathering a sufficient number of features is a vital requirement for classification tasks. However, increasing the number of features improves the classification abilities only to a certain point. This is called the curse of dimensionality. The curse of dimensionality is a common problem in machine learning caused by exponentially increasing errors with the number of features. A larger number of features requires a larger dataset, but because practically the number of training data is fixed, the classifier’s performance will drop after the number of features reaches a certain point, depending on the size of the dataset.

The excessive number of features also leads to other problems:

1. Overfitting. It is a condition when the model has learned so many random fluctuations and noise that it cannot learn from new data.
2. Too many features make each observation in the dataset equidistant from all others. However, if all data are approximately equidistant from each other, then all data look equal, and no significant predictions can be made.

Feature selection refers to several methods that resolve this fundamental problem by the dimensional reduction of unnecessary variables. From a set of features *F* = {*f*1, *f*2, …, *fn*}, feature selection methods define the ones that contribute the most to the learning ability. Feature reduction helps to improve the classifier’s learning abilities, reduces overfitting and training time, and removes unnecessary noise. 

Automatic feature selection is a popular method for the inclusion of brain data features, such as certain features of motor imagery [13] and features important for diagnosis using PET data [14], or for automated electroencephalography (EEG) data classification [15]. However, auditory illusion data have not been studied sufficiently, and there is no universal approach or strong basis for applying feature selection algorithms. Analyzing selected features that define octave illusion classification will contribute to the general understanding of auditory illusion mechanisms and, accordingly, the mental issues’ processes.

We used magnetoencephalography (MEG) data because, unlike EEG, it provides the origins of brain functional activity. Functional magnetic resonance imaging (fMRI), which simply measures blood flow instead of directly measuring the brain’s signals, also does not provide the necessary information. Moreover, the combination of MEG and the frequency-tagging method provides access to the contribution of each ear to the responses in the auditory cortices of each hemisphere. Therefore, we suggest that analyzing ASSR through MEG data will reveal the difference in auditory cortex activity between the auditory cortex of the ILL and non-ILL groups.

In this study, we aimed to find the most efficient union of the automatic selection method and machine learning method by comparing their various combinations. Considering the analyzed literature, to the best of our knowledge, this is the first study dedicated to the automatic feature extraction of octave illusion data for the classification of ILL and non-ILL groups.

## 2. Materials and Methods

### 2.1. Experimental Paradigm

This study involved MEG data of 17 male right-handed participants (9 ILL and 8 non-ILL) with a mean age and standard deviation of 21.4 ± 1.09 years. All participants were right-handed and had no history of otolaryngological or neurological disorders. All participants provided written consent after being informed of the nature of the study. This study was performed in accordance with the Declaration of Helsinki and was approved by the Research Ethics Committee of Tokyo Denki University.

MEG was recorded using a 306-channel whole-head-type brain magnetic field measurement device (VectorView 306, Elekta Neuromag, Neuromag, Helsinki, Finland). The brain magnetic field measurement device was installed in a magnetically shielded room, and the octave illusion tones were presented to the participants from the stimulation computer, after which the MEG was measured. After the analog-to-digital conversion of the measured MEG, the data were loaded into a computer at a sampling frequency of 1000 Hz.

Adobe Audition CS6 (Adobe Systems Incorporated) was used to generate tones. For a higher tone, the sound intensity was set to 3 dB, which is lower than the sound pressure level of the low tone.

### 2.2. Behavioral Testing

**Figure 4:**
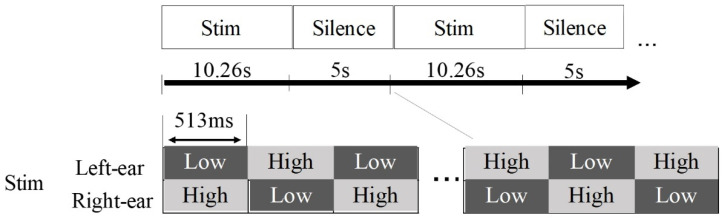
Experimental design. Audio stimuli had low (400 Hz) and high (800 Hz) tones, with a duration.

To classify the participants into ILL and non-ILL groups, we conducted a behavioral experiment in which each participant was equipped with headphones (E-A-RTONE 3A, Aearo Company Auditory Systems, Indianapolis, IN, USA) and presented with an octave illusion sound from a computer (ThinkPad Lenovo). Tones that were 513 ms long with modulation frequencies of 400 and 800 Hz (Figure 4) were played to the left and right ears, and each participant wrote on paper the perceptual pattern while the combination of the first stimulus scales and modulation frequencies of the left and right ears were changing. All participants listened to the sounds until they fully understood the perceptual pattern. As instructed, the participants wrote “Low” when they perceived a low tone, wrote “High” when they perceived a high tone, or left the space blank in the case of zero perception. 

The behavioral experiment results showed that the participants who alternately perceived the high and low tones from each ear were classified as the ILL group, and those who perceived the actual sounds were classified as the non-ILL group.

### 2.3. Preprocessing

Each participant’s session was cut to 1026 ms, one switch of 513 ms long tone, and averaged 96 times. All data were then subjected to bandpass filtering at 36–38 Hz. In the last preprocessing step, we applied source estimation to Heschl’s gyrus (primary auditory cortex).

In this way, one set of features consisted of 2052 features of ASSR values (1026 values of each ms from each left and right hemisphere). Because we only have data of 17 subjects, 2052 features for one sample was considered as an extremely large number, which can lead to overfitting. Therefore, we decided to use the ASSR signals only after 513 ms because the change of tones gives the most impact on ASSR amplitudes. This leads us to 1026 features for each subject. This number still can be considered large; however, since we do not have reliable information about which features differ between illusion and nonillusion groups, in order to not lose important features, we decided to use 513 features for each left and right hemisphere—1026 in total for each subject.

### 2.4. Machine Learning

Machine learning is a number of algorithms aimed to learn data, find specific patterns according to task, and make decisions without human intervention. There are three main categories of machine learning: supervised machine learning, which is characterized by using labeled data to train the model; unsupervised machine learning, which is characterized by analyzing and clustering unlabeled datasets; and reinforcement learning, which is based on rewarding and punishing the model according to its desired behavior. 

Since we have labeled data and we are interested in using the trained model with other similar data in the future, we used supervised machine learning in our study.

All algorithms require a set of related data to extract features that characterize the problem. The structure and quality of data is the most important factor for receiving reliable results of models’ performance. The more various and clean data, the more accurate the performance will be. Moreover, for any given task, some specific models can show better results than others. There are no exact rules on how to choose the best model for a task. It is necessary to test several algorithms to find the one that gives the most accurate results.

In our study, we had data of only 17 samples, which did not provide enough variety of data. This is why we focused on using several simple models with L2 regularization to avoid overfitting and compared the results to find the most optimal one.

In this study, we compared the results of four main classification approaches: logistic regression, random forest, support vector machine, and convolutional neural network.

#### 2.4.1. Logistic Regression

**Figure 5:**
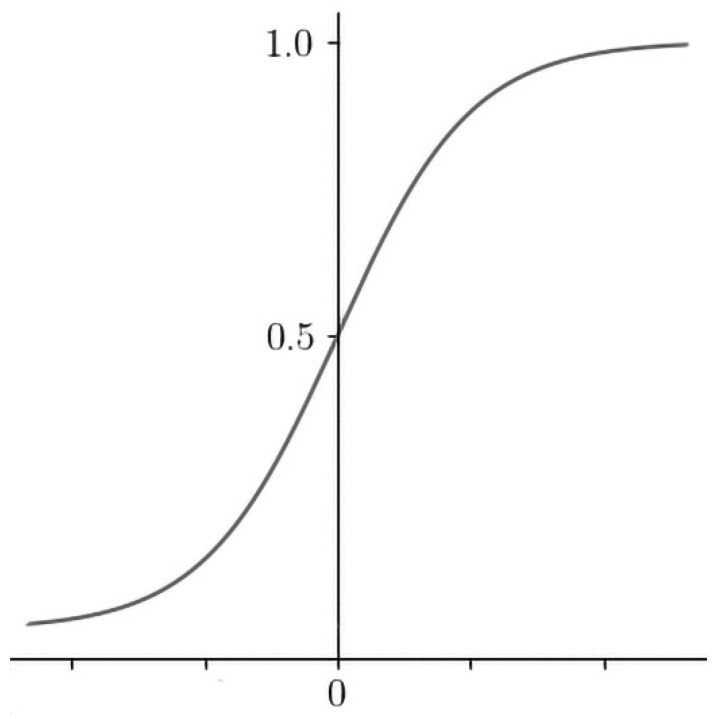
Logistic regression.

Logistic regression (LR) is a classification algorithm used to estimate binary values as true/false or 0/1 (Figure 5). The function quantifies the likelihood that a training sample point is correctly classified by the model. Therefore, the average for the entire training set indicates the probability that a random data point will be correctly classified by the system, regardless of the possible class. LR tries to maximize the mean of the data. For binary classification, the logistic regression model can be expressed as

(1) $$P\left(y\right)=1-\frac{1}{\;1+\exp\left(w\;x+b\right)}$$

where *P* is the probability, *y* is the outcome of interest, *w* is the weight, and *b* is a bias term [16]. LR is a simple, fast-training, feature-derivation classification method that gives good results on a small dataset with many features, which is the case in this study.

Since there is a literature gap in verified knowledge about octave illusion data features, in our study, to identify the dependencies between variables, we used a trial-and-error approach to choose the most accurate parameters for our model. The model with the following parameters showed the most accurate results for LR: gamma (inverse of the standard deviation) = 0.0001, C (inverse of regularization strength) = 1.0, L2 regularization.

#### 2.4.2. Random Forest

**Figure 6:**
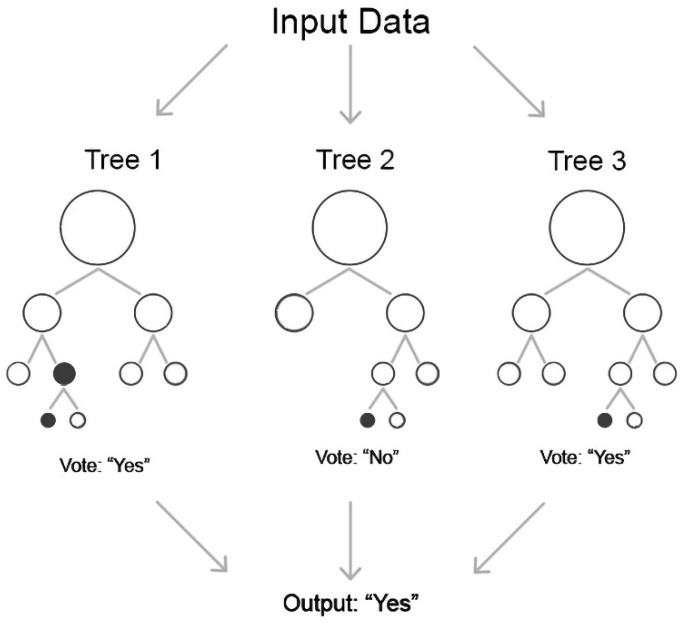
Random forest.

Random forest (RF) is an ensemble version of the decision tree algorithm. Each decision tree in the ensemble “votes” for a certain classification decision, and the prediction with the majority of voices “wins” (Figure 6). RF uses multiple trees to compute the majority of votes in the last leaf nodes to make a decision. Using decision trees, random forest models have resulted in significant improvements in prediction accuracy compared with a single tree by increasing the number of trees. In addition, each tree in the training set was randomly sampled without replacement. Each decision tree in the forest presents a simple structure in which the top node is considered the root of the tree that is recursively split at a series of decision nodes from the root until the decision node is reached [16]. Compared with other methods, RF is less prone to overfitting and works well with an automated feature interaction, making it a suitable method for classifying octave illusion data.

As with LR, to identify the dependencies between variables we used a trial-and-error approach to choose the most accurate parameters for our model. The model with the following parameters showed the most accurate results for RF: number estimators = 100, samples = 2, and the maximum number of features.

#### 2.4.3. Support Vector Machine

**Figure 7:**
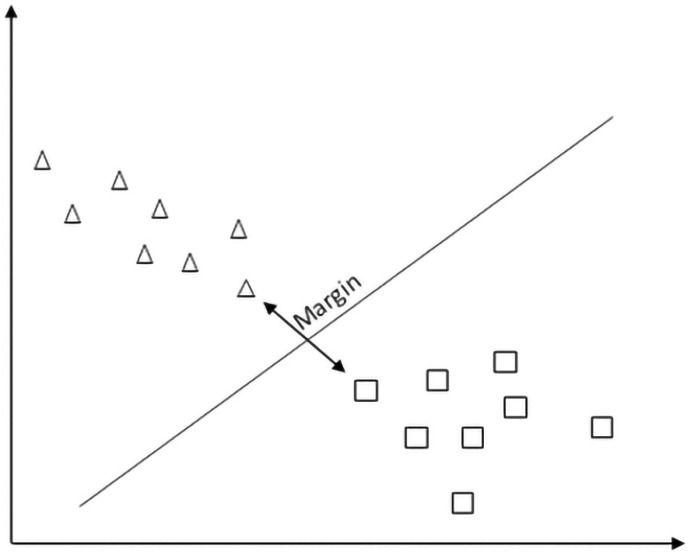
Support vector machine.

Support vector machine (SVM) is a two-class classification method that finds the optimal linear hyperplane in the feature space that maximally separates the target classes, saving space for misclassification (Figure 7). The common formula for the linear classifier is:

(2) $$f\left(x\right)=\overset{n}{\underset{i}{\sum }}\alpha_{i}k\left(x\;,x_{i}\right)+b$$

where α is the margin hyperplane, *x* and *x_i_* are separable classes, *k* is a kernel function, *b* is a linear parameter, and *i* = 1, 2, 3, …, *n* [17]. There could be an infinite number of hyperplanes separating classes, but because it is a two-dimensional space, any hyperplane will always have one dimension, which can be represented by a simple regression line. 

Although SVM, same as LR, shows good results on a small dataset with many features, unlike LR, it handles outliers better. 

Along with the approach for LR and RF, we again used a trial-and-error approach to choose the most accurate parameters for our model. The model with the following parameters showed the most accurate results for SVM: gamma = 0.0001, C = 1.0, L2 regularization.

#### 2.4.4. Convolutional Neural Network

Neural networks would be an effective method even for data with this high level of impurity; however, on a small dataset with many features, neural networks are more easily overfitted than other methods. Nevertheless, in order to explore possibilities of deep learning as well, we investigated the application of a convolutional neural network (CNN) for classification of octave illusion data. Although CNNs are commonly used for computer vision tasks, they have proved their efficiency in other fields, such as signal processing or medical applications. Furthermore, CNNs can be especially effective for biomedical data because they are tolerant to the input data transformations such as scaling or distortion and they can adapt to different input sizes [18].

Because our original dataset consists of 2D arrays of size 1026 × 2 (time/hemisphere), in order not to lose data, we could not use anything except 1D CNN structure, and to avoid overfitting, we used extremely simple CNN architecture of only three layers: convolutional, max-pooling, and fully connected (Table 1).

However, since it is impossible to establish exactly which features the CNN has used for its training and classification, in our study, we used the CNN as a tool to find that there is a difference in ASSR response between two groups and to compare its classification results with simpler methods of machine learning.

### 2.5. Feature Selection

In machine learning, gathering a sufficient number of features is a vital requirement for classification tasks. However, increasing the number of features improves the classification abilities only to a certain point. This is called the curse of dimensionality. The curse of dimensionality is a common problem in machine learning caused by exponentially increasing errors with the number of features. A larger number of features requires a larger dataset, but because practically the number of training data is fixed, the classifier’s performance will drop after the number of features reaches a certain point, depending on the size of the dataset [19]. Since in our study we have the dataset of only 17 subjects with 1026 features for each subject, using feature selection is necessary for effective classification of octave illusion and nonillusion data.

**Figure 8:**
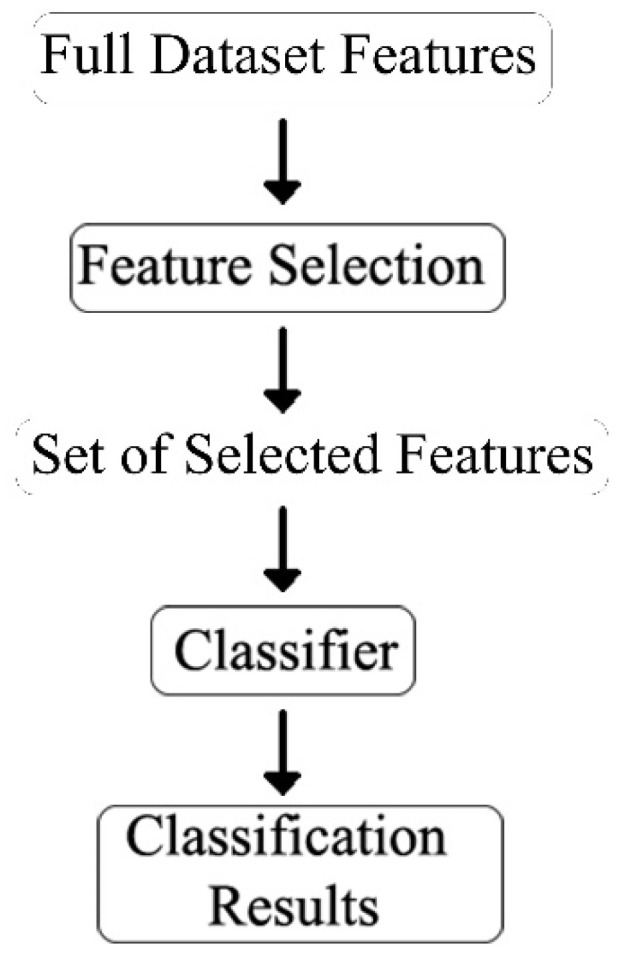
Feature selection process.

Feature selection should not be confused with feature extraction. Feature extraction creates a new set of features by mapping the original set of features. In contrast, feature selection takes a subset of the existing features without creating a new one. The overall feature selection process used in this study is shown in Figure 8.

In our study, we compared the results of four feature selection methods, which are univariate feature selection, recursive feature elimination, principal component analysis, and feature importance.

#### 2.5.1. Univariate Feature Selection

Univariate feature selection (US) is a method of selecting features that contribute the most to the classification using univariate statistical tests. It returns a ranked list of features based on different statistical scoring functions. The main characteristic of a univariate approach is that it does not consider the dependencies between the features, and in the end, features of the dataset are independent of each other [20].

#### 2.5.2. Recursive Feature Elimination

Recursive feature elimination (RFE) works by recursively removing values and uses the remaining attributes to build the model. First, the classifier is trained on the initial set of features, and the importance of each feature is written. The least important features are then cut from the features list. This procedure is recursively repeated until the desired number of quality features is reached [21].

#### 2.5.3. Principal Component Analysis

Principal component analysis (PCA) chooses variables based on the magnitude (from largest to smallest absolute values) of their coefficients. PCA is fast and easy to implement, but it does not count the potential multivariate nature of the data structure, which leads to the loss of potentially valuable features [21].

#### 2.5.4. Feature Importance

Feature importance (FI) (or variable importance) is a method to calculate scores for each feature for a given model. A feature is considered “important” if the accuracy of the model drops, and its change causes an increase in errors. However, a feature is considered “unimportant” if the shuffling of its values does not affect the accuracy of the model. There are several approaches to calculate the importance of features; in our study, we used an ensemble of decision trees (random forest) with mean decrease impurity. The algorithm randomly rearranges or shuffles one column of the validation dataset, leaving all other columns untouched [22]. It is a quick and easy-to-implement method with a tendency to prefer features with high cardinality, which is one of the important characteristics of our dataset.

## 3. Results

The main problem with using feature selection is its stochastic nature, which could lead to different results. To eliminate all possible ambiguities, each combination of machine learning and feature selection methods was run 10 times. Owing to the size of 17 of the entire dataset, we set the size of the training dataset to 11 (6 illusion and 5 nonillusion data), with the validation dataset of 4, and the test dataset to 6 (3 illusion and 3 nonillusion data) (Table 2). Again, based on the relatively small size of the training dataset (11 data in total), we focused on choosing the appropriate number of features.

First, we ran LR, RF, and SVM without using feature selection. Since we decided to use data from the time period between 513 and 1026 ms, we have 1026 features of ASSR signals from both the left and right hemispheres for the dataset of 17 subjects. To provide an understanding of used features, the short list of original features is shown in Table 3. Using the trial-and-error approach, the best parameters for each classifier are as follows:

- LR: gamma (inverse of the standard deviation) = 0.0001, C (inverse of regularization strength) = 1.0, L2 regularization.
- RF: number estimators = 100, samples = 2, maximum number of features.
- SVM: gamma = 0.0001, C = 1.0, L2 regularization.

The results for each algorithm are listed in Table 4.

Both LG and RF showed the same unsatisfactory results, with only 50% accuracy. In contrast, the SVM steadily showed 66.6% accuracy, which, considering the size of the dataset, can be called a satisfactory result.

In order to find the appropriate amount of features that will give stable classification results without losing too many data, we decided to remove the number of selected features gradually, by dividing it in half, and test the results. From the original dataset for machine learning of 1026, we stopped on datasets of 200, 30, and 40 features selected by each feature selection method. The results for 200 and 30 features, which showed some improvements, are presented in Table 5, Table 6, Table 7 and Table 8. Those steps were selected as turning points to choose a direction for increasing or decreasing the number of features. The dataset of 40 selected features (Table 9 and Table 10) showed the best and most stable results.

The results of the univariate feature selection for the dataset of 200 features are shown in Table 5. The method showed no improvement in combinations with LR and SVM but showed better results of RF: 66.6% instead of 50%. The results for RFE, PCA, and FI are shown in Table 6. None of the methods showed any difference from the original dataset.

The results of the univariate feature selection and feature importance for the dataset of 30 features are listed in Table 7. The method showed no improvement in combinations with LR and SVM from the original dataset, and the same RF results of 66.6% as for the dataset of 200 features. The results for RFE and PCA are shown in Table 9. It showed better results again of RF 66.6% instead of 50%. The results for other methods stayed the same.

The results of the univariate feature selection and feature importance for the dataset of 40 are listed in Table 8. Both methods showed no improvement in combination with LR, same results with RF, and better results of SVM with 75% accuracy. Although both methods showed the same accuracy results, US proved to be a more stable approach owing to its constant selection of the same features. Values selected by FI were different for each run, which is an expected behavior considering its stochastic nature. However, since we are interested in defining features of ASSR that contribute to octave illusion classification, a large variety in selection is unsatisfying data.

The results for both RFE and PCA showed no difference for LG, RF, and SVM and are listed in Table 10. As in the case of applying feature importance, we faced the problem with different sets of selected features after every run. Because the selected features were different every time and did not match even once after 10 runs, we created 10 sets of selected features for each RFE and PCA and ran them multiple times to obtain reliable results. The results shown are the majority of accurate results (7 out of 10) for all methods.

The dataset of 40 features received by using the US, which showed the best classification results aside from CNN, almost entirely consists of data from the left hemisphere. Datasets created by using RFE, PCA, and FI consist more of data from the right hemisphere, but the majority is left for the left one. Timecodes of those features are scattered along the time axis, and it is difficult to make a statement about when the difference between illusion and nonillusion groups happens exactly, but we can say that it takes place in the left hemisphere.

**Figure 9:**
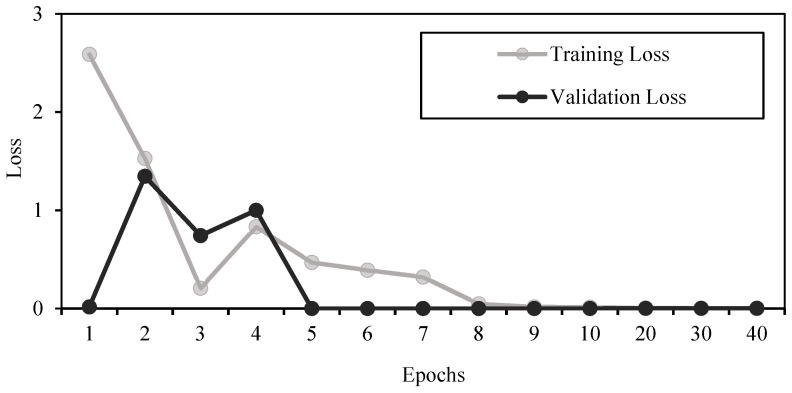
Training and validation losses.

Compared to using other machine learning methods with or without feature selection, applying deep CNN to the original dataset of features gave the best results of 100% accuracy, sensitivity, and specificity (Table 9 and Table 11). This made the used CNN structure the most efficient tool to classify octave illusion and nonillusion data using ASSR, but it does not contribute to our knowledge about exactly which features of ASSR make the difference between the two groups. Since there are no false positive or false negative results and the training and validation losses are both small after epoch 10 (Figure 9), we can say that features were extracted successfully and no overfitting had happened.

## 4. Discussion

**Figure 10:**
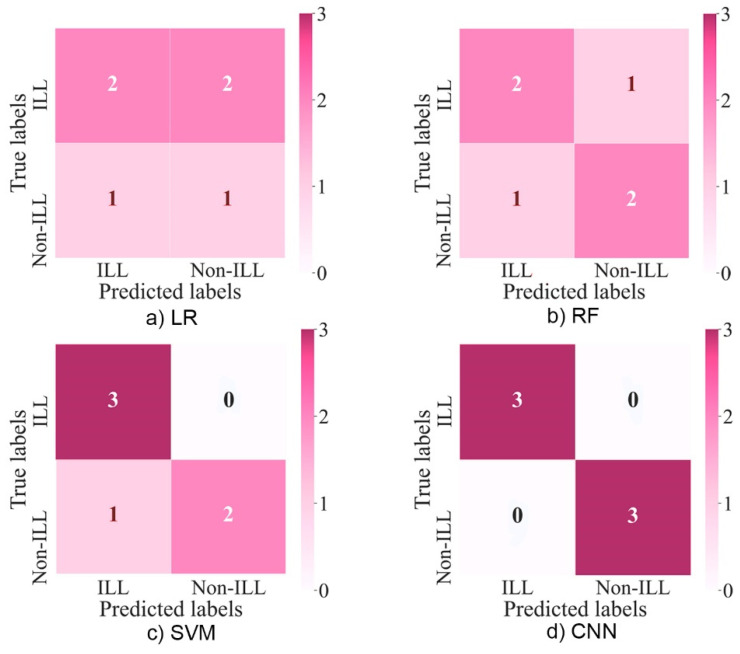
Confusion matrix for four methods.

In this study, we aimed to find the most efficient combination of feature selection and machine learning methods for classifying octave illusion data. Machine learning has been widely used for the classification of various brain data, from classifying brain–computer interface (BCI) data to decoding MEG signal processing. ASSR signals are quite difficult to define owing to their small amplitudes and high levels of brain noise. The combination of SVM as a machine learning algorithm with univariate selection and feature importance as feature selection methods showed the highest classification results with 75% accuracy, 100% sensitivity, and 66.6% specificity (Table 10), which, considering the small size of the training dataset, are satisfactory results. Applying CNN gives even better results with 100% accuracy, 100% sensitivity, and 100% specificity, which makes it the best classification method (Figure 10).

However, considering the big picture, we are not interested in simple classification of illusion and nonillusion data, but in obtaining information about exactly which ASSR values differentiate those two groups to obtain a deeper understanding of the auditory illusion mechanism. Since the FI, due to its stochastic nature, gives various results and requires several runs to average it, and the US in its turn always presents the same results, the combination of SVM with the US is preferable.

Univariate selection is the only feature selection method that provides almost the same set of ASSR features in every run. Using this method, we received the set of features that most clearly define the difference between octave illusion and nonillusion groups, which will help in constructing the classification tool for octave illusion and nonillusion data. Since this set almost entirely consists of data from the left hemisphere, we suggest that for the right-handed group of people, the mechanisms that cause the octave illusion are lying there. However, since no dependencies were found between these features, at this moment, it is difficult to define the pattern of auditory cortex activity for octave illusion and nonillusion groups.

In addition to using the developed machine learning methods for the classification of octave illusion and nonillusion data, information about selected features can be used to understand the underlying mechanisms of auditory illusions, which can contribute to managing mental diseases. In the future, we plan to build a universal tool for the classification of various types of auditory illusions based on the differences in the ASSR signals.

## Figures and Tables

**Table 1:** CNN architecture.

Layer	Kernel Size	Filter	Stride
Convolutional	1 × 1	4	1
Max-Pooling	1 × 2	-	4
Fully Connected	-	-	-

**Table 2:** Dataset.

Parameters	Dataset
Stimuli (low tone/high tone) (Hz)	400/800
Number of participants (ILL/non-ILL)	17 (9/8)
Training data (ILL/non-ILL)	11 (16/5)
Validation data (ILL/non-ILL)	6 (3/3)
Test data (ILL/non-ILL)	6 (3/3)

**Table 3:** Short list of original features.

Label	LH_513	LH_514	LH_515	RH_513	RH_514	RH_515
NILL	7.29 × 10^−13^	4.68 × 10^−13^	1.86 × 10^−13^	6.84 × 10^−12^	6.84 × 10^−12^	6.47 × 10^−12^
NILL	3.26 × 10^−12^	3.37 × 10^−12^	3.30 × 10^−12^	2.60 × 10^−12^	1.21 × 10^−12^	2.63 × 10^−13^
ILL	4.50 × 10^−12^	3.45 × 10^−12^	2.24 × 10^−12^	8.76 × 10^−12^	2.38 × 10^−12^	4.73 × 10^−12^
NILL	6.21 × 10^−12^	5.19 × 10^−12^	3.87 × 10^−12^	4.05 × 10^−13^	3.62 × 10^−13^	1.12 × 10^−12^
ILL	2.59 × 10^−12^	1.89 × 10^−12^	1.09 × 10^−12^	4.45 × 10^−13^	1.97 × 10^−12^	3.38 × 10^−12^
ILL	2.48 × 10^−12^	3.09 × 10^−12^	3.53 × 10^−12^	1.82 × 10^−12^	1.35 × 10^−12^	8.09 × 10^−13^
ILL	2.58 × 10^−12^	3.28 × 10^−12^	3.81 × 10^−12^	3.81 × 10^−12^	3.41 × 10^−12^	2.82 × 10^−12^
ILL	2.46 × 10^−13^	4.62 × 10^−13^	6.56 × 10^−13^	1.45 × 10^−12^	2.33 × 10^−12^	3.07 × 10^−12^
ILL	2.71 × 10^−12^	2.56 × 10^−12^	2.28 × 10^−12^	3.17 × 10^−12^	2.66 × 10^−12^	2.01 × 10^−12^
ILL	3.20 × 10^−12^	4.44 × 10^−12^	5.45 × 10^−12^	1.35 × 10^−12^	1.75 × 10^−12^	2.05 × 10^−12^
ILL	2.60 × 10^−12^	1.98 × 10^−12^	1.26 × 10^−12^	4.98 × 10^−12^	5.11 × 10^−12^	4.97 × 10^−12^
NILL	8.77 × 10^−13^	1.50 × 10^−12^	2.04 × 10^−12^	6.66 × 10^−13^	5.40 × 10^−13^	3.94 × 10^−13^
NILL	2.45 × 10^−12^	2.41 × 10^−12^	2.24 × 10^−12^	3.74 × 10^−12^	4.63 × 10^−12^	5.28 × 10^−12^
NILL	3.56 × 10^−12^	4.02 × 10^−12^	4.25 × 10^−12^	5.04 × 10^−12^	4.22 × 10^−12^	3.16 × 10^−12^
NILL	3.25 × 10^−12^	3.24 × 10^−12^	3.05 × 10^−12^	3.96 × 10^−12^	5.27 × 10^−12^	6.29 × 10^−12^
ILL	6.08 × 10^−12^	6.07 × 10^−12^	5.71 × 10^−12^	9.50 × 10^−12^	9.54 × 10^−12^	9.08 × 10^−12^
NILL	1.71 × 10^−12^	1.60 × 10^−12^	1.40 × 10^−12^	1.09 × 10^−12^	1.07 × 10^−12^	1.01 × 10^−12^

LH is left hemisphere; RH is right hemisphere; 513, 514, and 515 are time codes in ms; ILL is illusion data; NILL is nonillusion data.

**Table 4:** Classification results for machine learning and CNN methods.

Method	TP	TN	FP	FN
CNN	3	3	0	0
LR	2	1	2	1
RF	1	2	1	2
SVM	2	2	1	1

TP: true positive, TN: true negative, FP: false positive, FN: false negative.

**Table 5:** Classification results for machine learning methods with US of 200 features.

Method	TP	TN	FP	FN
LR US/FI	2	1	2	1
RF US/FI	2	2	1	1
SVM US/FI	2	2	1	1

TP: true positive, TN: true negative, FP: false positive, FN: false negative.

**Table 6:** Classification results for machine learning methods with RFE/PCA/FI of 200 features.

Method	TP	TN	FP	FN
LR RFE/PCA	2	1	2	1
RF RFE/PCA	1	2	1	2
SVM RFE/PCA	2	2	1	1

TP: true positive, TN: true negative, FP: false positive, FN: false negative.

**Table 7:** Classification results for machine learning methods with US/FI of 30 features.

Method	TP	TN	FP	FN
LR US/FI	2	1	2	1
RF US/FI	2	2	1	1
SVM US/FI	2	2	1	1

TP: true positive, TN: true negative, FP: false positive, FN: false negative.

**Table 8:** Classification results for machine learning methods with RFE/PCA of 30 features.

Method	TP	TN	FP	FN
LR RFE/PCA	2	1	2	1
RF RFE/PCA	2	2	1	1
SVM RFE/PCA	2	2	1	1

TP: true positive, TN: true negative, FP: false positive, FN: false negative.

**Table 9:** Classification results for machine learning methods with US/FI of 40 features.

Method	TP	TN	FP	FN
LR RFE/PCA	2	1	2	1
RF RFE/PCA	2	2	1	1
SVM RFE/PCA	3	2	1	0

TP: true positive, TN: true negative, FP: false positive, FN: false negative.

**Table 10:** Classification results for machine learning methods with RFE/PCA of 40 features.

Method	TP	TN	FP	FN
LR RFE/PCA	2	1	2	1
RF RFE/PCA	2	2	1	1
SVM RFE/PCA	2	2	1	1

TP: true positive, TN: true negative, FP: false positive, FN: false negative.

**Table 11:** Overall classification results.

Method	Accuracy (%)	Sensitivity (%)	Specificity (%)
CNN	100	100	100
LR	50	66.6	33.3
RF	50	33.3	66.6
SVM	66.6	66.6	66.6
LR US/FI (40 features)	50	66.6	33.3
RF US/FI (40 features)	66.6	66.6	66.6
SVM US/FI (40 features)	75	100	66.6
LR RFE/PCA (40 features)	50	66.6	33.3
RF RFE/PCA (40 features)	66.6	66.6	66.6
SVM RFE/PCA (40 features)	66.6	66.6	66.6
